# A New Protocol for Homogeneity Testing in Feed Mill Concentrate Rations

**DOI:** 10.3390/ani16010046

**Published:** 2025-12-24

**Authors:** Raphaela Cenci Vidal, Edenio Detmann, Marcia de Oliveira Franco, Daiana Francisca Quirino, Marcos Inácio Marcondes, Alex Lopes da Silva, Laiane Silva, Polyana Pizzi Rotta

**Affiliations:** 1 PH Controle de Qualidade, Brasília 70774-100, DF, Brazil; raphaelacenci@gmail.com (R.C.V.); laiane.trabalhos@gmail.com (L.S.); 2 Department of Animal Science, Universidade Federal de Viçosa, Viçosa 36570-900, MG, Brazil; alex.lopes@ufv.br (A.L.d.S.); polyana.rotta@ufv.br (P.P.R.); 3 Production Systems, Natural Resources Institute Finland (Luke), 31600 Jokioinen, Finland; marcia.franco@luke.fi; 4 Technology Park of Viçosa—TecnoPARQ, Universidade Federal de Viçosa, Viçosa 36576-400, MG, Brazil; daiana.f.quirino@gmail.com; 5 William H. Miner Agricultural Research Institute, Chazy, NY 12921, USA; mmarcondes@whminer.com; 6 College of Agronomy and Veterinary, Universidade de Brasília, Brasília 70910-900, DF, Brazil

**Keywords:** feed mill, homogeneity test, magnesium, potassium, sampling protocol

## Abstract

To ensure an accurate homogeneity test, animal feed manufacturers should apply standardised techniques and procedures, which aim to guarantee the product quality. Our objective was to propose a new protocol for performing concentrate ration homogeneity tests in commercial feed mills, based on three main points: the suitability of different minerals as markers; establishing a simplified and reliable sampling protocol; and developing a simplified statistical approach for evaluating marker dispersion among increments. Four horizontal commercially available mixers were used. Increments were collected sequentially during the mixer-emptying time over five consecutive days, totalling 200 increments, in which nine potential mineral markers were evaluated. The minerals potassium (K) and magnesium (Mg) were considered suitable, as they simultaneously exhibited low variability among batches, among mixers, and among increments. Based on the variation patterns of K and Mg, the new protocol for homogeneity testing in feed mill concentrate rations is based on the following additional key points: two increments must be collected per ration batch, taken during the mixer-emptying operation—specifically at the second and eight tenths of the mixer-emptying time, and a concentrate ration mixture is considered homogeneous when the ratio between the highest and lowest marker contents in the increments is lower than 1.26.

## Simple Summary

Ensuring that animal feed is well mixed is essential for producing safe and consistent rations in feed mills. To find the best way to check whether the feed is properly mixed, we carried out a study in a commercial feed mill, tested different markers, and proposed a sampling method and statistical approach for a ration homogeneity test. We found that the minerals potassium and magnesium work very well as markers of how evenly the feed is mixed. When mixing is performed correctly, the amount of these minerals changes very little from one mixer, batch, or sample to another. Because of this low variation, they are reliable for identifying when the mixing process is not working as it should. Based on these results, we developed a simple protocol to evaluate the quality of the mixing process: two samples are taken from each batch—one early and one later during the mixer’s discharge. The levels of potassium or magnesium in these samples are then measured. If the ratio between the highest and lowest mineral levels is smaller than 1.26, the ration is considered properly mixed. If the difference is larger, it suggests a mixing problem, meaning that the process or the equipment needs to be checked.

## 1. Introduction

Regarding a ration or feed, homogeneity is a theoretical concept that implies that the material is composed of perfectly identical fragments—identical in every possible aspect, including size, composition, density, surface morphology, electrostatic charge [1]. Clearly, such a condition can never be achieved in practice, and all rations or feeds must therefore be considered heterogeneous materials. Consequently, the practical application of term homogeneity should be interpreted as the minimisation of heterogeneity.

According to the Theory of Sampling (TOS), there are two types of heterogeneity, which are complementary and inclusive. Compositional heterogeneity refers to intrinsic differences in composition among individual fragments or constituents. It is evident that any ration produced by mixing different ingredients is compositionally heterogeneous, and no mixing operation can alter this type of heterogeneity [1,2,3]. In simple terms, rations are expected to exhibit compositional heterogeneity because they are formed from different ingredients.

On the other hand, distributional heterogeneity refers to the heterogeneity imparted to the whole due to the spatial arrangement of the different groups (or constituents) within it [1,4]. In other words, distributional heterogeneity reflects the non-random distribution of particles, arising mainly from the effects of gravity on particles that differ in density, size, and shape, which promotes particle grouping and segregation [2]. This is the key aspect of heterogeneity to consider when producing rations or feeds. Mixing has a deterministic effect on these between-group differences: as mixing progresses, the virtual groups become increasingly similar [1]. Therefore, distributional heterogeneity—or, conversely, homogeneity (hereafter referred to simply as heterogeneity or homogeneity)—is central to monitoring the quality of rations provided to animals or marketed commercially.

In this sense, mixture homogeneity tests verify whether each feed or ration fraction consumed by the animal contains the nutrient levels declared on the product label [5]. As previously noted, the mixing operation is critical to ration production [6], because the concentrations of nutrients and other components in each aliquot of a batch should be similar [7]. However, perfect mixing is unattainable due to several factors—such as the order in which ingredients are added to the mixer, ingredient moisture, particle size and density, and electrostatic charges—which can promote segregation [7,8,9].

To ensure an accurate homogeneity test, feed manufacturers should apply standardised techniques and procedures to assess product quality [9]. Such tests rely on three key elements: the use of a marker capable of being homogeneously dispersed throughout the ration; a sampling protocol that specifies how increments are collected and how many are taken from each batch; and a statistical evaluation of the dispersion of marker concentration across theses increments.

The markers used are generally ingredients present at low contents in the mixture, such as amino acids, minerals, microtracers (e.g., coloured iron particles), or antimicrobials [10]. Although specific amino acids have been recommended as markers, their analysis is costly and often inaccessible to commercial feed mills [8]. Minerals, on the other hand, are widely used in feed formulations and are the most common markers for evaluating mixing efficiency, as their analytical quantification is relatively simple and inexpensive [11,12]. However, some minerals may occur at high concentrations in feeds, which may compromise their effectiveness as markers of ration mixing [5] and reduce the reliability of mixture diagnostics. Frequently, homogeneity tests in commercial feed mills in Brazil are performed based on zinc (Zn) and, or manganese (Mn) concentrations. However, to our knowledge, there is still no information in the literature identifying which markers are most effective for assessing ration-mixing homogeneity.

As previously noted, a crucial aspect in evaluating the effectiveness of a ration-mixing process is the sampling protocol [13]. Collecting appropriate increments ensures the representativeness of the analytical samples [14], which in turn provides reliable information on marker concentration and dispersion, thereby allowing robust homogeneity diagnostics. Currently, sampling protocols based on 7 to 10 increments per ration batch are commonly used for batches of up to 2.5 tonnes [9,15], although this approach lacks strong empirical and scientific support. These recommendations do not account for interactions with marker characteristics and also some important aspects of TOS, such as sampling dimensionality. Consequently, such recommendations lack proper validation under commercial feed-mill conditions.

The dispersion of the marker concentration across increments is generally assessed using Pearson’s coefficient of variation (CV) [16]. As widely recommended, an effective mixing process should exhibit low CV values, which indicate low heterogeneity and high uniformity within or between batches [8,17]. Under this criterion, the CV of marker distribution among increments collected within a ration batch should be below 10%, indicating effective mixture of the ration ingredients [18,19]. When the CV value falls between 10 and 15%, the mixing process is generally considered acceptable. Conversely, a CV above 20% indicate a non-uniform mixing process, suggesting that either the mixing operation or the equipment should be re-evaluated [20]. However, those overall CV ranges appear largely subjective, as they do not account for the influence of marker characteristics, sampling procedures, or the intrinsic properties of the ration.

Considering the three key elements of a homogeneity test and all their potential influences and interactions, it is evident that further research is needed to develop methods and standardise processes that ensure a robust evaluation of ration-mixing efficiency. Such evaluation should encompass both repeatability (i.e., within the same mixer) and reproducibility (i.e., between mixers and, or feed mills). Therefore, our objective was to propose a new protocol for performing concentrate-ration homogeneity tests in commercial feed mills, based on three main key points: 1. the suitability of different minerals as markers; 2. the establishment of a simplified and reliable sampling protocol; and 3. the development of a simplified statistical approach for evaluating marker dispersion across increments.

## 2. Materials and Methods

The experiment was conducted in a commercial feed mill in Luziânia, Goiás, Brazil. No ethics committee approval was required, as the study involved only the analysis of animal feed under routine factory conditions, with no interference with normal operations. Furthermore, employees were not instructed to change or modify their daily management practices.

### 2.1. Equipment, Mixing Procedures, and Sampling

Four horizontal helical mixers were used: two with a capacity of 1000 kg (Wadin, MetaGril, Goiatuba, Goiás, Brazil) operating at 32 revolutions per minute (rpm), and two with a capacity of 800 kg (Discometal, Indústria e Comércio de Máquinas e Metais Perfurador, Goiânia, Goiás, Brazil) operating at 30 rpm. To evaluate mixing quality, a standard concentrate ration for lactating dairy cows was produced in each mixer for five consecutive days. The ration was composed of ground maize, soybean meal, limestone, urea, sodium chloride, dicalcium phosphate, and a mineral-vitamin mix (i.e., flowers of sulphur, magnesium oxide, iron sulphate, zinc oxide, copper sulphate, manganese monoxide, calcium iodate, cobalt sulphate, sodium selenite, vitamin A, vitamin D_3_, vitamin E, and monensin), formulated to contain 240 g crude protein/kg as fed. The feed ingredient composition of the concentrate ration was (as fed) was: ground maize—626 g/kg, soybean meal—300 g/kg, limestone—40 g/kg, urea—17 g/kg, sodium chloride—12 g/kg, dicalcium phosphate—2 g/kg, and mineral-vitamin mix—3 g/kg.

**Figure 1:**
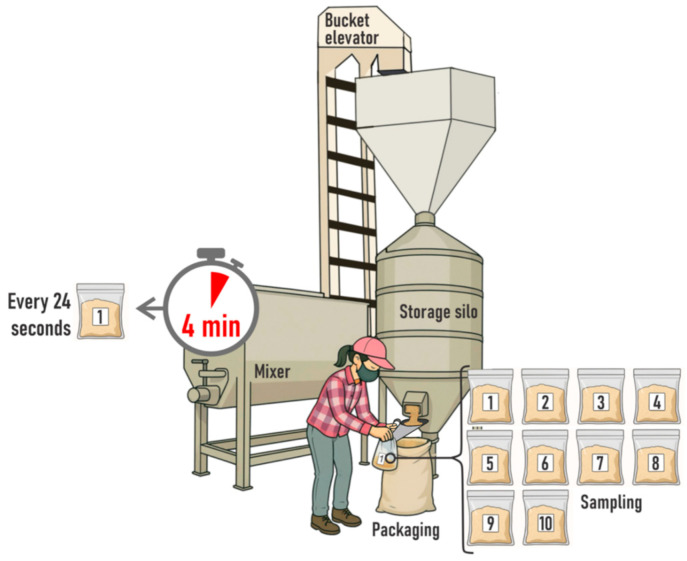
Experimental design and sampling protocol for evaluating commercial horizontal helical mixers.

The ingredients were weighed and added to the mixers according to the AAFCO [21] recommendations. The major macro-ingredient was added first (i.e., ground maize), followed by the minor ingredients (i.e., limestone, urea, sodium chloride, dicalcium phosphate, and mineral-vitamin mix), with soybean meal added last. Mixing time was set at 4 min for all mixers (Figure 1). All mixers were operated strictly in accordance with the manufacturers’ instructions to ensure an optimised mixing process. All mixer operation procedures were in accordance with feed manufacturing techniques described by AAFCO [21].

The mixer container and storage silo are structures with a three-dimensional manifestation of distributional heterogeneity, which makes any sampling protocol difficult to design and costly to implement. The fewer the dimensions involved, the easier it is to solve the sampling problem associated with a batch or lot [22]. Therefore, collecting increments during the emptying operation effectively reduces the batch to a one-dimensional object (i.e., a flowing stream) [1,22]. Accordingly, our sampling protocol was based on collecting increments during mixer emptying at the point of packaging (Figure 1). This procedure represents a cross-cut sampling approach, in which each increment consists of a cross-section of the flowing stream, ensuring that all particles have an equal probability of being selected. Although the principle is fundamentally spatial in nature, its practical application necessarily involves a temporal component, because material flows during mixer emptying exhibit temporal variability in particle distribution. Thus, multiple cross-cuts must be taken at different moments during discharge. Each increment represents a spatially correct (i.e., cross-cut) snapshot of the stream at a given instant, and the combination of increments collected over time captures the full distributional heterogeneity of the batch [22]. In this sense, the cross-cut sampling is spatial in design but temporal in execution, as representativeness can only be achieved by a sequence of spatially correct increments collected throughout the duration of material flow. Following these principles, the emptying time of each mixer was estimated and then divided into ten equal intervals (i.e., a total emptying time of 4 min resulted in ten 24 s intervals). Hereafter, these intervals are referred to as tenths of the mixer emptying time.

In summary, an increment was defined as an individual portion of material collected by a single sampling operation from the decision unit (i.e., the ration batch; [14,15]). Accordingly, starting at the onset of mixer emptying, an increment of the concentrate mixture was taken at each tenth of the emptying time directly from the outlet of the filling silo, resulting in a total of ten increments per batch. Each increment weighed approximately 200 g, was individually packed in a clean plastic bag, and sealed immediately (Figure 1). At the ending of the sampling procedure, a total of 200 increments were collected (4 mixers × 5 days × 1 batch per day per mixer × 10 increments per batch).

### 2.2. Laboratory Analysis

After the trial period, all increments were sent to the laboratory for mineral analysis to evaluate potential markers of mixing efficiency and homogeneity. Nine potential mineral markers were quantified: calcium (Ca), phosphorus (P), potassium (K), sodium (Na), magnesium (Mg), iron (Fe), Zn, copper (Cu), and Mn. Analyses were performed according to the methods described by Sarruge and Haag [23] and Detmann et al. [24]. Briefly, a mineral solution was produced for each increment using nitro-perchloric digestion [25]. Mineral contents were then quantified using colorimetry (P; V-M5, BEL Engineering, Milano, Italy), flame photometry (Na and K; Corning 400, Corning Medical, Suzano, SP, Brazil), and atomic absorption spectrophotometry (Ca, Mg, Fe, Zn, Cu, and Mn; GBC Avanta Σ, Scientific Equipment, Braeside, Victoria, Australia) [23,24]. The limits of detection (mg/L of reading solution) for the minerals were: Ca—0.126; P—0.017, K—0.034, Na—0.024, Mg—0.033, Fe—0.006, Zn—0.010, Cu—0.002, and Mn—0.006. Mineral contents were expressed on an as-fed basis to avoid bias introduced by additional dry matter quantification.

### 2.3. Statistical Analysis

Initially, results of each mineral marker were subjected to analysis of variance according to the model:

(1) $$Y_{ijk}=\mu +M_{i}+B_{\left(i\right)j}+S_{k}+\varepsilon_{ijk}$$

where Y_ijk_ is the mineral content in the increment taken from batch j in mixer i at time k (i.e., k-th tenth of the mixer emptying time); μ is the general constant (fixed effect); M_i_ is the random effect of mixer i, assumed to be NIID (0, σ^2^_M_); B_(i)j_ is the random effect of batch j nested within the mixer i, assumed to be NIID (0, σ^2^_B/M_); S_k_ is the fixed effect of the increment collected at k-th tenth of the mixer emptying time; and ε_ijk_ is the random error, assumed to be NIID (0, σ^2^_ε_).

The increments were considered repeated measurements during the mixer emptying process. Therefore, the residual (co)variance matrix was modelled assuming heterogeneous variances for each time and covariances between times [i.e., an unstructured (co)variance matrix], allowing the assessment of marker variability throughout the mixer emptying process. All variance components were estimated using the restricted maximum likelihood method [26].

The variances obtained from the statistical model Equation (1) were standardised relative to the average marker content across all sampling times as follows:

(2) $${RSD}_{M}=\frac{\sqrt{{\hat{\sigma }}_{M}^{2}}}{\hat{\mu }}\times 100$$

(3) $${RSD}_{B}=\frac{\sqrt{{\hat{\sigma }}_{B/M}^{2}}}{\hat{\mu }}\times 100$$

(4) $${RSD}_{Ik}=\frac{\sqrt{{\hat{\sigma }}_{\varepsilon_{k}}^{2}}}{\hat{\mu }}\times 100$$

where RSD_M_ and RSD_B_ are the relative and standardised standard deviations between mixers and batches, respectively (%); RSD_Ik_ is the relative and standardised standard deviation among increments taken at the k-th tenth of the mixer emptying time (%); $\hat{\mu }$ is the estimated mean mineral marker content; and ${\hat{\sigma }}_{M}^{2}$, ${\hat{\sigma }}_{B/M}^{2}$ and ${\hat{\sigma }}_{\varepsilon_{k}}^{2}$ are the estimated variance components corresponding to mixers, batches nested within mixers, and residual variance at k-th tenth of the mixer emptying time, respectively.

The maximum range of mineral content was estimated as:

(5) $$R_{max}=\max\left({\hat{\mu }}_{k}\right)-\min\left({\hat{\mu }}_{k}\right)$$

where R_max_ is the maximum range of the mineral or marker content, and ${\hat{\mu }}_{k}$ is the average content of the marker at the k-th tenth of the mixer emptying time.

The statistical significance of R_max_ was assessed using the total Studentised range, considering a null hypothesis based on a parametric value of 0 and a two-tailed alternative hypothesis (α = 0.05).

The estimated R_max_ was then standardised as:

(6) $${RR}_{max}=\frac{R_{max}}{\hat{\mu }}\times 100$$

where RR_max_ is the relative or standardised R_max_ (%).

To enable graphical comparison among mineral markers, their contents at different tenths of the mixer emptying time were standardised so that all minerals shared the same overall mean (i.e., 100). Standard errors of the means at each time point were also expressed as percentages, as follows:

(7) $${RMV}_{k}=\frac{{\hat{\mu }}_{k}}{\hat{\mu }}\times 100$$

(8) $${VI}_{k}=\pm \frac{s_{{\hat{\mu }}_{k}}}{{\hat{\mu }}_{k}}\times 100$$

where RMV_k_ is the relative or standardised mean content at the k-th tenth of the mixer emptying time (%), VI_k_ is the variation index, defined as the relative standard error of the mean content at the k-th tenth of the mixer emptying time (%), $\hat{\mu }$ is the overall mean mineral marker content, ${\hat{\mu }}_{k}$ is the mean mineral marker content at the k-th tenth of the mixer emptying time, and $s_{{\hat{\mu }}_{k}}$ is the standard error of ${\hat{\mu }}_{k}$.

All statistical analyses were performed using the GLIMMIX procedure of SAS 9.4 (α = 0.05).

## 3. Results and Discussion

The results obtained in our study are based on a set of assumptions necessary for their correct interpretation. First, we assumed that the entire mixing process was carried out optimally. Moreover, we adopted the assumption that the final concentrate ration mixture can be considered an infinite-element material. Thus, the ration mixture would consist of a practically infinite number of indistinguishable elements that cannot be individually identified [14]. From this, the ration mixture is interpreted as a continuous and homogeneous matrix or medium (i.e., with minimal distributional heterogeneity). In contrast, the different mineral markers would theoretically be considered as a finite-element material [14], consisting of individually identifiable elements whose distribution within the ration mixture would be discernible by an adequate analytical method. Therefore, the marker concentration in the increments could conceptually be interpreted as the frequency of occurrence of these finite elements per unit of mass of a continuous medium formed by infinite elements. Based on these assumptions, the ration mixture is considered homogeneous, and any deviation from homogeneity indicated by a marker would reflect its intrinsic limitation to be adequately diluted in the matrix (i.e., to show a homogeneous frequency of occurrence). Therefore, all results presented in this study and their respective interpretations are based on the aforementioned assumptions.

Overall, the mineral markers exhibited different patterns of variability between mixers and batches (Table 1). Considering the previously presented assumptions, a high variability among mixers and batches would indicate limitations of a marker for evaluating the reproducibility and repeatability of the mixing process, respectively. In this sense, it is reasonable to expect that an ideal marker should indicate variability estimates tending toward zero for a suitable mixing process. In the case of inter-mixer variability (i.e., an approach to the mixing process reproducibility), near-zero variability was observed for P, K, Mg, Cu, and Mn.

Furthermore, a value tending towards zero for an ideal marker is expected to indicate inter-batch variability within the same mixer, which could allow for a direct association with the repeatability of the mixing process. In this case, low or near-zero variability was observed for Ca, Na, P, K, Mg, and Mn.

In addition to the variability between mixers and batches, the importance of low variability between increments collected under similar sampling conditions is also emphasised. Assuming that the medium (i.e., the ration matrix) is continuous and homogeneous, as previously highlighted, high variability in mineral marker content between increments could indicate limitations in the dispersibility of the marker in the ration mixture, which could lead to a false diagnosis of a non-homogeneous mixture. In our study, the sampler and sampling procedures were identical for all mixers and batches: they were performed by the same sampler and using the same sampling device, and were compared within the same sampling moment (Table 1). In this sense, the lowest variability between increments was observed for K and Mg.

**Figure 2:**
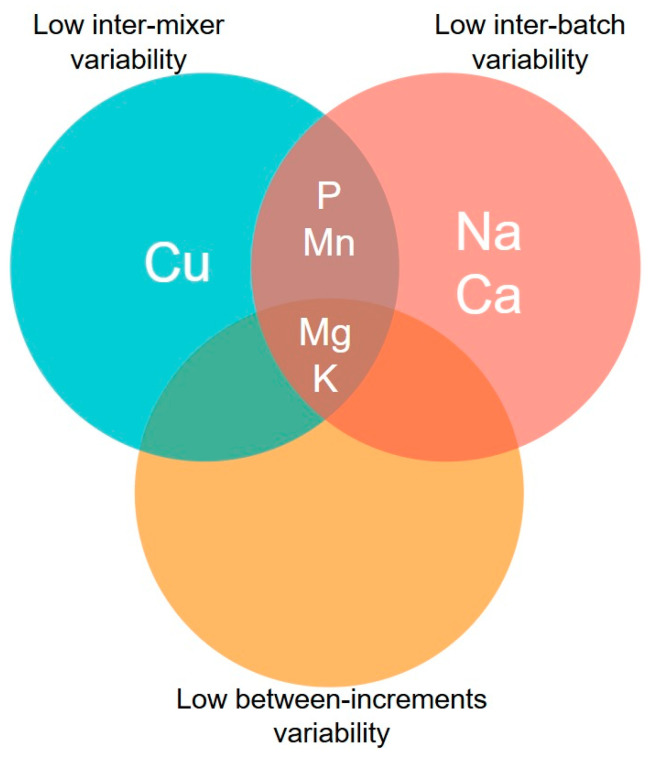
Venn diagram for the marker variability considering the three different dimensions of variability: inter-mixer, inter-batch, and between-increment variability (for details, see Table 1).

Therefore, an intersection of the results described above can be made based on the three evaluated dimensions (i.e., between mixers, between batches, and between increments). Under this perspective, only K and Mg fulfilled all desired characteristics (Figure 2).

**Figure 3:**
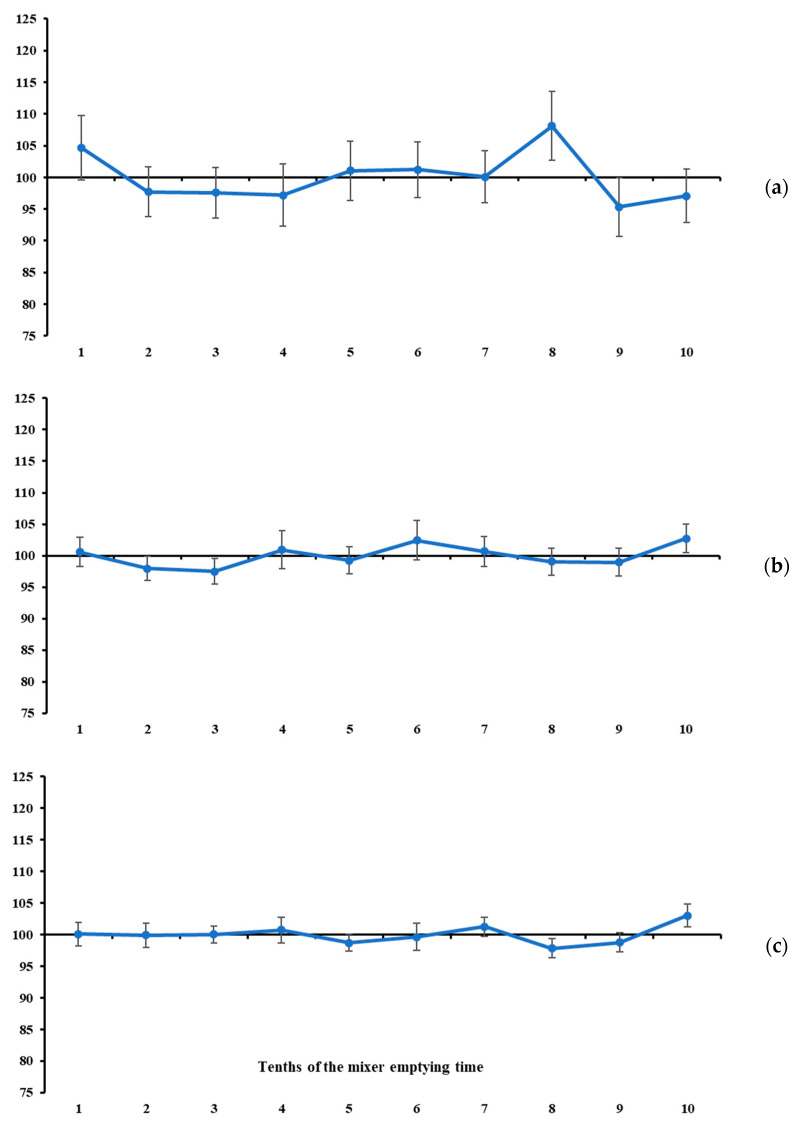
Standardised mean values of Ca (**a**), P (**b**), and K (**c**) contents across increments taken at different tenths of mixer emptying time. Vertical bars represent the variation index (refer to Equations (7) and (8)).

**Figure 4:**
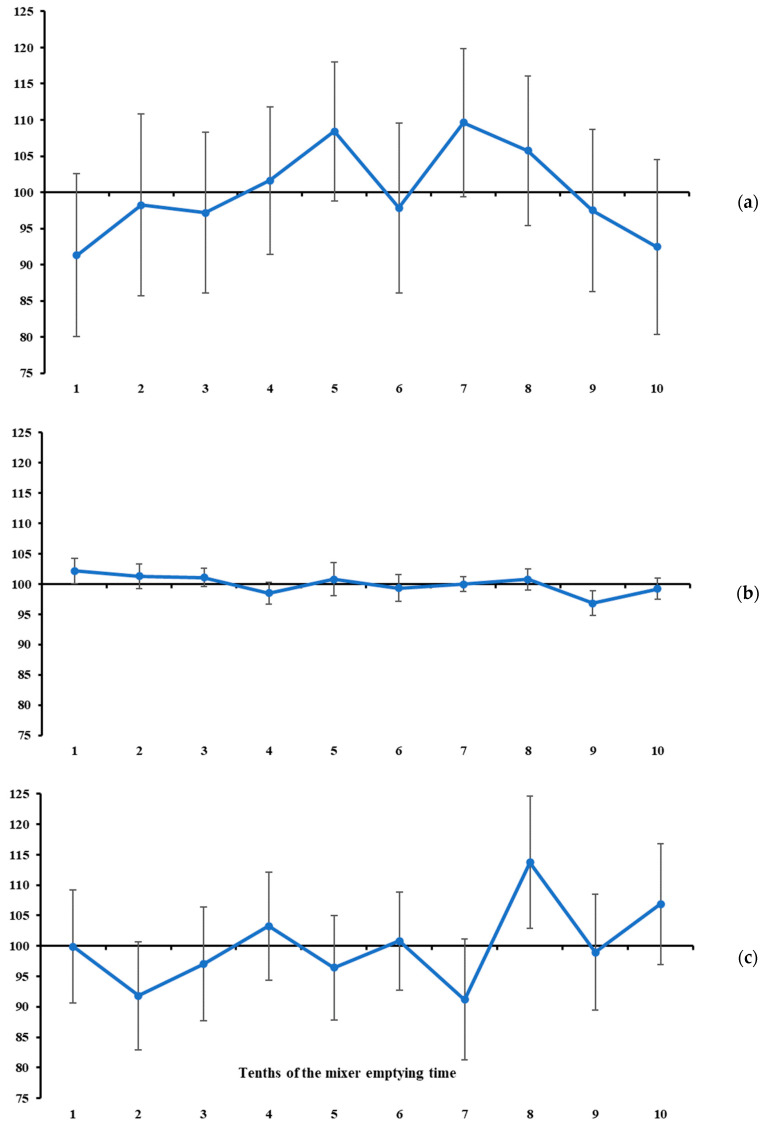
Standardised mean values of Na (**a**), Mg (**b**), and Fe (**c**) contents across increments taken at different tenths of mixer emptying time. Vertical bars represent the variation index (refer to Equations (7) and (8)).

**Figure 5:**
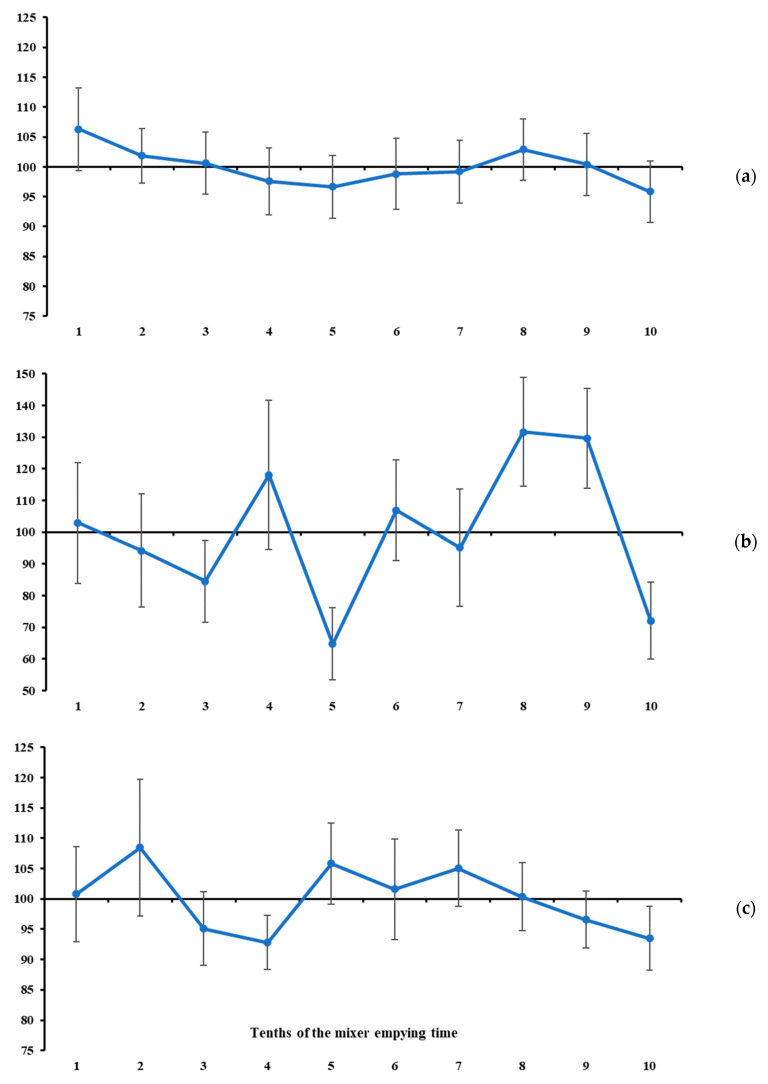
Standardised mean values of Zn (**a**), Cu (**b**), and Mn (**c**) contents across increments taken at different tenths of mixer emptying time. Vertical bars represent the variation index (refer to Equations (7) and (8)).

In our study, the measures of location of the mineral marker contents were used as complementary information to the measures of variability. In general, none of the total ranges of mineral marker contents differed from zero (*p* > 0.08, Table 2, Figure 3, Figure 4 and Figure 5). At first glance, this result would indicate that the maximum oscillation among the contents evaluated at different tenths of mixer emptying time is null, which would lead us to conclude that all markers indicate a homogeneous mixing process. However, this pattern does not suggest that marker selection should be discretionary. On the contrary, it gives greater weight to variability measures in the sampler’s decision-making. If the mineral markers are similarly accurate in indicating homogeneity, the choice should rely on the one that behaves more precisely (see the previously presented assumptions). In this sense, and in line with the statements above, the lowest total ranges and variabilities were observed for K (Figure 3c) and Mg (Figure 4b). Despite the narrow total range, P is not recommended due to the high between-increment variability compared with the two mineral markers mentioned above (Table 1).

To provide an adequate diagnosis of ration-mixing efficiency, a marker must indicate both the accuracy and precision of the process and have a feasible and inexpensive analytical method [8]. Moreover, it should exhibit physical properties comparable to those of other feed components, so it can disperse easily in the medium [13,27]. In theory, markers for homogeneity tests may be internal, when intrinsic to the ration feed components, or external, when not naturally present. However, when evaluating ration mixing efficiency, a marker can also be simultaneously internal and external. This hybrid classification applies to minerals that are naturally present in feeds, usually at low concentrations, but also added through mineral premixes. This is the case for Mg, suggested as one of the two potential markers in this study. Magnesium is predominantly external (i.e., most Mg comes from mineral premix), but also internal, as feeds naturally contain Mg. On the other hand, K tends to be exclusively internal, as it is abundant in plant-based feeds, such as maize grain and soybean meal [28,29,30].

When a marker is predominantly internal to feed components, another relevant characteristic must be highlighted: its content across feed ingredients should be as different as possible. An internal marker whose contents across ration feed components are similar is not capable of diagnosing mixing efficiency. In this case, regardless of the degree of mixing, the final content tends to be similar, providing insufficient sensitivity to detect deviation from ideal mixing. Considering this, markers with predominantly external characteristics seem to be slightly more reliable, as their contribution to the final ration derives mainly from mineral premixes. Therefore, Mg seems more advantageous than K for most commercial concentrate rations. However, in our case, using K did not constitute a limitation, as its average content differs substantially among the ingredients used (maize grain, 3.5 ± 0.5 g/kg dry matter; soybean meal, 20.2 ± 1.0 g/kg dry matter; [30]).

A common recommendation for a ration homogeneity test is to take 7 to 10 increments from each ration batch of up to 2.5 tonnes [1,15], with the marker content being individually evaluated using an appropriate analytical method [13]. However, it seems that the literature is not entirely clear regarding a standard sampling procedure to guide the sampler on how increments should be collected. Moreover, when considering a set of increments within a ration batch, the CV of the marker content among increments has been widely used to diagnose the adequacy of the ration-mixing process. However, the interpretation of CV is partially subjective, as it depends on the marker, analytical uncertainty, and the chemical and physical properties of the feed ingredients [13]. This means that any adopted CV range [18,19,20] can be seen as discretionary and partially subjective. Nevertheless, a robust protocol to evaluate ration mixing efficiency should be as objective as possible to ensure reproducibility across samplers, feasibility under different industrial conditions, and its use as a reliable tool for comparisons among batches, mixers, and feed mills.

Under the assumptions presented at the beginning of this section, and considering that the recommended markers (i.e., K and Mg) exhibited a stable pattern throughout the mixer’s emptying time (Figure 3 and Figure 4), it seems that 7 to 10 increments are not necessary to diagnose mixing efficiency for batches of up to 2.5 tonnes. From this, we here propose an objective protocol to evaluate ration mixing efficiency based on two increments collected independently during the mixer emptying operation (i.e., sampling from a flowing stream—a one-dimension object [22]). Our main objective is to develop an evaluation protocol with a sound scientific basis and maximum objectivity and reproducibility.

Our method is based on three simple assumptions: 1. the marker used is suitable for representing mixing efficiency, 2. sampling is performed on a one-dimensional object by taking increments during the mixer-emptying operation, and 3. the two increments to be evaluated are independent and collected at different and appropriate times during the mixer-emptying operation.

Consider X_1_ and X_2_ as the marker contents in increments 1 and 2, respectively, collected during the mixer emptying operation. Thereafter, it is assumed that X_1_ represents the higher marker content of the two increments. The values of X_1_ and X_2_ can be standardised based on their mean content, as follows:

(9) $$\bar{X}=\frac{X_{1}+X_{2}}{2}$$

(10) $$P_{1}=\frac{X_{1}}{\bar{X}}\times 100$$

(11) $$P_{2}=\frac{X_{2}}{\bar{X}}\times 100$$

Equations (9)–(11) show that the mean of the standardised values equals 100 arbitrary units. Therefore, the value 100 corresponds to the mean and to the midpoint between P_1_ and P_2_. The following properties of the difference between P_1_ and P_2_ can be derived from these statements:

(12) $$d=\left|P_{1}-P_{2}\right|=|\frac{X_{1}-X_{2}}{\overset{̿}{X}}\times 100|$$

(13) $$\left|P_{1}-100\right|=\left|P_{2}-100\right|=\frac{d}{2}$$

where d denotes the difference between the standardised values (i.e., P_1_ and P_2_).

From the standardisation, the following assumption is adopted: the marker, when expressed in arbitrary units (Equations (10) and (11)), can be considered as a finite-element material (i.e., a discrete material) distributed along a medium that corresponds to an infinite-element material (i.e., the concentrate mixture is assumed to be a continuous medium). Under this assumption, the marker presents an expected frequency of occurrence equal to 100 arbitrary units per unit mass of the continuous medium (Equations (9)–(11)). Therefore, once the marker is assumed to be a finite-element (discrete) material, its standardised content per unit of medium mass can be interpreted as a frequency of occurrence, whose numerical pattern follows a χ^2^ distribution. Thus, the expected frequency of marker occurrence (i.e., the finite or discrete element) is 100 arbitrary units per unit mass of medium, whereas the observed frequencies are represented by the standardised values P_1_ and P_2_. In an ideal ration-mixing process, the observed frequencies P_1_ and P_2_ should be equal and converge to 100. From this, the evaluation of the mixing efficiency becomes analogous to a hypothesis test, in which the null hypothesis is that the observed frequencies P_1_ and P_2_ are equal to the expected frequency of 100.

Using the Neyman-Pearson lemma, the null hypothesis described above would be accepted if the calculated χ^2^ statistic does not exceed the critical χ^2^ value. Consequently, for a homogeneous ration mixture, the following condition must be satisfied:

(14) $$\chi_{calculated}^{2}<\chi_{critical}^{2}$$

(15) $$\sum_{i=1}^{2}\frac{{\left(f_{observed}-f_{expected}\right)}^{2}}{f_{expected}}<\chi_{critical}^{2}\Rightarrow \frac{{\left(P_{1}-100\right)}^{2}}{100}+\frac{{\left(P_{2}-100\right)}^{2}}{100}<\chi_{critical}^{2}$$

By applying the property defined in Equation (13), we obtain:

(16) $$\frac{{\left(d/2\right)}^{2}}{100}+\frac{{\left(d/2\right)}^{2}}{100}<\chi_{critical}^{2}\Rightarrow d<\sqrt{200\times \chi_{critical}^{2}}$$

The critical χ^2^ statistic is defined by the number of degrees of freedom—which is equal to 1 in the present situation—and by the α value, which represents the maximum tolerated probability of an undue rejection of the null hypothesis. Here, the rejection of the null hypothesis implies concluding that the mixture cannot be considered homogeneous. An analogous pair of statistical errors can be derived from this reasoning: a type I error consists of identifying a homogeneous ration mixture as non-homogeneous, whereas a type II error consists of identifying a non-homogeneous ration mixture as homogeneous. In feed mill operations, a type II error is clearly more hazardous for commercial purposes and feed safety. As such, an α value of 0.10 is suggested to provide stricter control over type II error. Considering that the region corresponding to null hypothesis rejection is located unilaterally in the right tail of χ^2^ distribution, we have:

(17) $$\chi_{critical}^{2}=\chi_{\alpha \left(d.f.\right)}^{2}=\chi_{0.10\left(1\right)}^{2}≅2.71$$

By applying (16) in (15), we obtain:

(18) $$d<\sqrt{200\times 2.71}≅23.28$$

If we consider the d value obtained in (17) as the maximum tolerable range between P_1_ and P_2_, and assume that 100 represents the midpoint between these values, we obtain, under ideal ration-mixing conditions:

(19) $$\max P_{1}=100+\frac{d}{2}=100+\frac{23.28}{2}=111.64$$

(20) $$\min P_{2}=100-\frac{d}{2}=100-\frac{23.28}{2}=88.36$$

Based on these values, the initial standardisation process is reverted using Equations (9) and (10):

(21) $$\max P_{1}=\frac{\max X_{1}}{\bar{X}}\times 100\Rightarrow \max X_{1}=\frac{\max P_{1}\times \bar{X}}{100}=\frac{111.64\times \bar{X}}{100}=1.1164\times \bar{X}$$

(22) $$\min P_{2}=\frac{\min X_{2}}{\bar{X}}\times 100\Rightarrow \min X_{2}=\frac{\min P_{2}\times \bar{X}}{100}=\frac{88.36\times \bar{X}}{100}=0.8836\times \bar{X}$$

Calculating the ratio between the maximum permissible value of X_1_ and the minimum permissible value of X_2_ gives the maximum critical ratio (MCR), which allows conclusions to be drawn about the sufficient homogeneity of a mixture:

(23) $$MCR=\frac{\max X_{1}}{\min X_{2}}=\frac{1.1164\times \bar{X}}{0.8836\times \bar{X}}=\frac{1.1164}{0.8836}≅1.26$$

Thus, if X_1_ and X_2_ are the marker concentrations in increments 1 and 2, collected independently at appropriate times during mixer emptying process, with X_1_ > X_2_, a ration mixture is considered homogeneous (α = 0.10) if:

(24) $$\frac{X_{1}}{X_{2}}<1.26$$

All markers evaluated in this study, with the exception of Cu, satisfied this criterion (Table 2), but the lowest ratios were observed for K and Mg, reinforcing their suitability as ideal markers. It is noteworthy that P also exhibited a low ratio; however, it was previously disqualified as an ideal marker due to its high variability between increments (Table 1).

However, one aspect of the protocol still needs to be defined, namely the timing of collection of the two increments to be used in the evaluation described above. The timing of occurrence of maximum and minimum contents was highly variable across all markers (Table 2). However, when considering only K and Mg, their contents were stable throughout the mixer-emptying operation (Figure 3 and Figure 4). Therefore, at least in theory, the timing of increment collection would not influence the statistical diagnosis. Nevertheless, a free-choice recommendation could lead to a lack of robustness and reproducibility of the protocol, and, in some cases, increments could be collected at very close time points of the emptying operation, which could result in a false diagnosis of homogeneity. To avoid this potential drawback, we suggest a protocol based on collecting increments at the second and eighth tenths of the mixer emptying time. These specific tenths represent symmetrical time points relative to the beginning and end, as well as to the midpoint, of the emptying operation, and are sufficiently spaced to ensure sensitivity for detecting differences in marker content. For example, if the total emptying time of a ration mixer is 600 s, the increments would be collected at 120 (i.e., 2 × 60 s) and 480 (i.e., 8 × 60 s) seconds from the beginning of the emptying operation. Both are symmetrical with respect to the start and end of the emptying operation (i.e., equally distant from 0 and 600 s, respectively) and are also symmetrical with respect to the midpoint of the emptying time (i.e., equally distant from 300 s).

In summary, the proposed protocol uses K or Mg as markers for concentrate ration homogeneity testing. Two increments per ration batch are used to evaluate marker contents in the ration mixture, collected at the second and eight tenths of the mixer-emptying time. For the ration mixing operation to be considered adequate, the ratio between the highest and lowest marker contents in the increments must be lower than 1.26 (Equation (24)). Otherwise, the concentrate ration cannot be considered properly mixed, and either the mixing process or the equipment should be re-evaluated. One of the main strengths of this protocol is that it is based on a one-dimensional sampling procedure, which minimises the general sampling issues associated with three-dimensional objects [22]. Therefore, the protocol proposed herein cannot be applied to a three-dimensional object, such as a mixer compartment or a storage silo. Conversely, the required number of increments may be influenced by the batch size. For this reason, the present protocol should not be used when the batch size exceeds 2.5 tonnes, in accordance with the upper limit prescribed by ISO [15].

## 4. Conclusions

From the investigations performed, we propose a new protocol for homogeneity testing in feed mill concentrate rations, which is based on the following key points: 1. potassium and magnesium are used as markers; 2. two increments (i.e., samples) are collected per ration batch, taken during the mixer-emptying operation, specifically at the second and eight tenths of the mixer-emptying time; and 3. a concentrate-ration mixture is considered homogeneous when the ratio between the highest and lowest marker contents in the increments is lower than 1.26.

## Figures and Tables

**Table 1:** Relative standard deviations (%) of mineral markers across mixers, batches, and sampling increments collected at different tenths of the mixer emptying time in a ration mixing process.

	Mineral Marker								
Source of Variation ^1^	Ca	P	K	Na	Mg	Fe	Zn	Cu	Mn
Between mixers	5.4	0.0	0.0	17.5	0.0	12.9	6.4	0.0	0.0
Between batches	0.4	2.3	0.3	0.9	2.0	3.0	6.2	16.2	2.1
Between increments									
1	20.4	10.0	8.1	23.8	9.5	29.7	28.8	86.2	35.3
2	12.2	8.1	8.5	39.0	9.1	21.9	13.8	73.1	54.6
3	12.4	8.6	6.0	28.0	6.6	28.3	17.4	46.2	25.7
4	17.4	13.5	9.0	24.4	7.6	28.9	18.8	123.4	18.3
5	17.4	9.2	6.0	24.9	12.2	23.0	16.2	28.8	31.6
6	15.8	14.3	9.7	33.1	9.5	21.6	21.2	74.2	37.6
7	14.1	10.6	7.1	31.4	5.2	28.3	17.1	76.9	29.7
8	23.3	9.2	6.8	29.3	7.6	47.4	17.7	99.9	25.2
9	15.4	9.5	6.6	28.9	8.5	30.6	17.2	89.8	20.1
10	13.8	10.4	8.3	31.1	7.5	37.6	15.6	35.8	22.0

^1^ For details, report to Equations (1)–(4).

**Table 2:** Descriptive statistics of mineral marker contents in the ration mixing process.

	Mineral Marker								
Item	Ca ^1^	P ^1^	K ^1^	Na ^1^	Mg ^2^	Fe ^2^	Zn ^2^	Cu ^2^	Mn ^2^
Overall mean	17.3	3.44	9.57	4.64	0.246	175	109	30.7	54.5
Maximum mean	18.7	3.53	9.86	5.08	0.251	199	115	40.4	59.1
Position of the maximum mean ^3^	8	10	10	7	1	8	1	8	2
Minimum mean	16.5	3.35	9.37	4.24	0.238	160	104	19.9	50.6
Position of the minimum mean ^3^	9	3	8	1	9	7	10	5	4
Total range	2.2	0.18	0.49	0.84	0.013	39	11	20.5	8.5
*p*-value	0.523	0.190	0.651	0.168	0.357	0.690	0.327	0.085	0.866
Standardised total range (%)	12.8	5.3	5.2	18.3	5.3	22.6	10.5	66.9	15.7
Maximum/Minimum	1.13	1.05	1.05	1.20	1.05	1.24	1.11	2.03	1.17

^1^ g/kg as fed. ^2^ mg/kg as fed. ^3^ It indicates the tenth of mixer emptying time. For details, refer to Figure 2, Figure 3 and Figure 4.

## Data Availability

Readers may request the corresponding author to provide data supporting the findings of this study under reasonable circumstances.
